# Streamlining land surface model Initialization: Automated data retrieval for VELMA using HMS REST API and GDAL

**DOI:** 10.1016/j.envsoft.2025.106492

**Published:** 2025-05-08

**Authors:** Kar’retta Venable, John M. Johnston, Stephen D. LeDuc

**Affiliations:** a US Environmental Protection Agency (USEPA), Office of Research and Development, Center for Environmental Measurement and Modeling, Ecosystem Processes Division, Landscape and Aquatic Systems Modeling Branch, 960 College Station Road, Athens, GA, 30605, USA; b USEPA Center for Public Health and Environmental Assessment, Office of Research and Development, Research Triangle Park, NC, 27711, USA

**Keywords:** Modeling workflow, HMS, VELMA, Jupyter notebook, Open hydrology

## Abstract

Continuous monitoring data required for performing environmental model simulations using gridded land surface models (LSMs) are often difficult to obtain and manage, making the modeling process challenging and prone to error. In response, this study focuses on automated retrieval and processing of digital elevation models (DEMs from Google Earth Engine (GEE)), meteorologic drivers of hydrology, and surface runoff time series data, using the Visualizing Ecosystem and Land Management Assessment (VELMA) model as a case study. Our automation methodology is accomplished using the USEPA’s Hydrologic Micro Services (HMS) Representation State Transfer (REST) application programming interface (API) and Geospatial Data Abstraction Library (GDAL) with Python. This workflow provides greater efficiency, minimizes data preparation time, reduces manual processing errors, and provides a reusable methodology for use in other modeling studies. With this innovation, users of VELMA and other gridded LSMs will be able to initialize simulations more efficiently, improving their operational capabilities.

## 1. Overview

### 1.1. Introduction

In environmental modeling, acquisition and curation of the copious amount of data to assess hazards and risks to public health requires extensive time and knowledge to enable researchers and other stakeholders to apply models that assess environmental impacts. For example, in gridded models, it can often take a tedious amount of time and a high degree of expert knowledge to apply the correct initialization data and preprocessing methodology. Concomitantly, however, many modeling efforts are data limited, presenting problems with model calibration and validation (Santos et al., 2013). To address data-poor regions, interpolation methods can be used to fill missing data in addition to machine learning techniques, and statistical fitting (Reichstein et al., 2019). To increase precision analysis and model predictions, development of a synergistic system which enables dynamic logistics, uncertainty characterization, and collection of data is desirable (Ouyang et al., 2007). An optimal retrieval process for hydrologic models’ initialization encompasses data collection like a dynamic data driven application system (DDDAS) approach (Ouyang et al., 2013). The novelty of this methodology enables the frequency, acquisition, and preprocessing for data required while developing records which are reproducible and repetitive for copious amounts of data for multiple delineated outlets of interest.

We used a DDDAS approach to create a novel method for the acquisition and preprocessing of data for a gridded model. To accomplish the goal of a DDDAS structure for hydrologic and land surface modeling, we used the United States Environmental Protection Agency’s (USEPA) Hydrologic Micro Services (HMS) and Google’s Earth Engine (GEE) to provide initialization data within the USEPA’s Visualizing Ecosystem and Land Management Assessment (VELMA) model. To test our DDDAS in VELMA, we performed a case study that addresses pre- and post-wildfire impacts on water quality associated with the Hayman wildfire of 2002 (Venable et al., 2024). Specifically, with VELMA, we simulate and analyze ecohydrological impacts within subbasins and Hydrologic Unit Codes (HUC). Since limited spatial and temporal observed data were available for a true DDDAS in our study region, we are utilizing modeled data to satisfy VELMA’s initialization requirements for the model. However, identification of required initialization data across the entire area of interest or other locations across the US presents fundamental issues critical to calibrate the model.

The associated impacts of the estimation of nutrient dynamics, plant productivity, water quality, and ecosystem health vary due to regional and subbasins water budgets. We were tasked with utilizing aggregated modeled data and satellite retrievals to initialize, analyze, and preprocess the environmental and ecohydrological conditions which impair post-fire water quality. Due to limited long-term records, inadequate identification of these connections across different temporal scales presents issues to categorize conditions within watershed boundaries (Golden et al., 2014). The issue with these data retrievals and initialization inputs requires translation between GeoTIFFs, rasters, and flat files, like the American Standard Code for Information Interchange (ASCII) (Oak Ridge National Laboratory, n.d.) files, requiring accurate comparisons between each of these data formats. Our methodology for this automated data acquisition methodology streamlines data acquisition, preprocessing, and analysis for VELMA and other gridded LSMs.

### 1.2. Research focus

This research focuses on automation methods for retrieving the digital elevation model (DEM), weather drivers (WD), and surface runoff across the United States (US) for a particular area of interest (AOI) to initialize the USEPA’s VELMA model and other gridded land surface models (LSMs). Streamlining gridded hydrometeorological data products promotes spatiotemporal comparisons between models at user-defined scales and are critical to hydrologic modeling (Nandi et al., 2024). These automation techniques enable users, programmatically and non-programmatically, to define spatial and temporal data collection for any area of interest within the contiguous US, promoting reductions in data assimilation time while accelerating calibration and validation of simulations. The data collection methodology creates the ability for other researchers to overcome the problem of reproducibility, a necessity for scientific advancement (Baker, 2016; Choi et al., 2021). Advancements in programming languages like Python, ease in the development of curation of scripts and environmental data management with Findability, Accessibility, Interoperability, and Reusability (FAIR) principles (Samourkasidis et al., 2019). Our goal is to provide a reproducible workflow for researchers and decision makers unfamiliar with programming and scripts, to enable users to easily retrieve large batches of initialization data. Improving data acquisition time and sources will enhance modeling and calibration efforts in VELMA or other LSMs. Here, we focus on the streamlined workflow developed, the deployment tools used, and the results across the AOI. Prior calibration metrics for the Hayman Fire AOI can be obtained from the LANDIS (LANDscape DIsturbance and Succession)-VELMA wildfire case study (Venable et al., 2024).

## 2. Background

### 2.1. VELMA

The USEPA’s VELMA (Abdelnour et al., 2011, 2013), provides grid-based catchment scale ecohydrological analysis in response to landuse change and biomass harvests or clearcuts. The flexibility in domain, grid cell size, landuse changes, initialization data, and biomass representation make VELMA an ideal vehicle for exploration of wildfire impacts on water quality and streamflow. Prior initialization methods for VELMA (Abdelnour et al.) and other LSMs require multiple data retrieval locations and methods for capturing the digital elevation model (DEM), weather drivers, and surface runoff to accurately represent spatial and temporal environmental conditions. To obtain initialization data for VELMA, it takes expertise in resolving the gridded data indices to latitude and longitude coordinates with the use of licensed geospatial software (McKane et al., 2014) and can develop errors of improperly matched spatial objects limiting retrieval efficiency (Yang et al., 2024). Provided methods for VELMA or other LSMs initialization of data aggregates can propagate human-made errors and promote additional time to accurately collect, organize, and utilize within model runs.

### 2.2. HMS

USEPA (Parmar et al., 2018) provides transparent modeling workflows and environmental data provisioning services related to hydro-informatics. This multimedia platform offers Findability, Accessibility, Interoperability, and Reusability (FAIR) practices while improving efficiency in hydrologic and environmental modeling within the US. These webservices retrieve data through online scripts and are utilized in additional components to adequately characterize watershed dynamics for HUCs. Data aggregates available from a variety of sources through HMS inclusive of rainfall, temperature, solar radiation, soil moisture, surface and subsurface streamflow, and evapotranspiration are available in a diverse set of timescales, output formats, and provide visual representation through interactive plots for data viewing. Outside of the web interface, HMS offers a Representation State Transfer (REST) Application Programming Interface (API), which provides example scripts for each of its data retrievals and workflow components to interact with client-server communications improving flexibility for data acquisition. However, HMS currently doesn’t allow autonomous retrievals of multiple reaches or coordinates unless within the same stream network.

### 2.3. GEE

Over the past decade, GEE has provided global open access for researchers to investigate environmental datasets enabling temporal geospatial exploration. Through the web-based Code Editor, API interaction with these datasets enable researchers to access a suite of client libraries through Java Script and Python wrappers to visualize data and develop customized user applications for exploratory data analysis. These curated datasets examine spatial and temporal issues of humanity including biomass impacts, water quality and quantity and environmental degredation through coupled cloud computing and remote sensing methodologies (Gorelick et al., 2017). An exceedingly copious amount of remotely sensed and observational data from US Government and International agencies are available for researchers to interpret but are constrained to coding language syntax and programming methodologies.

### 2.4. Case study

**Fig. 1.**
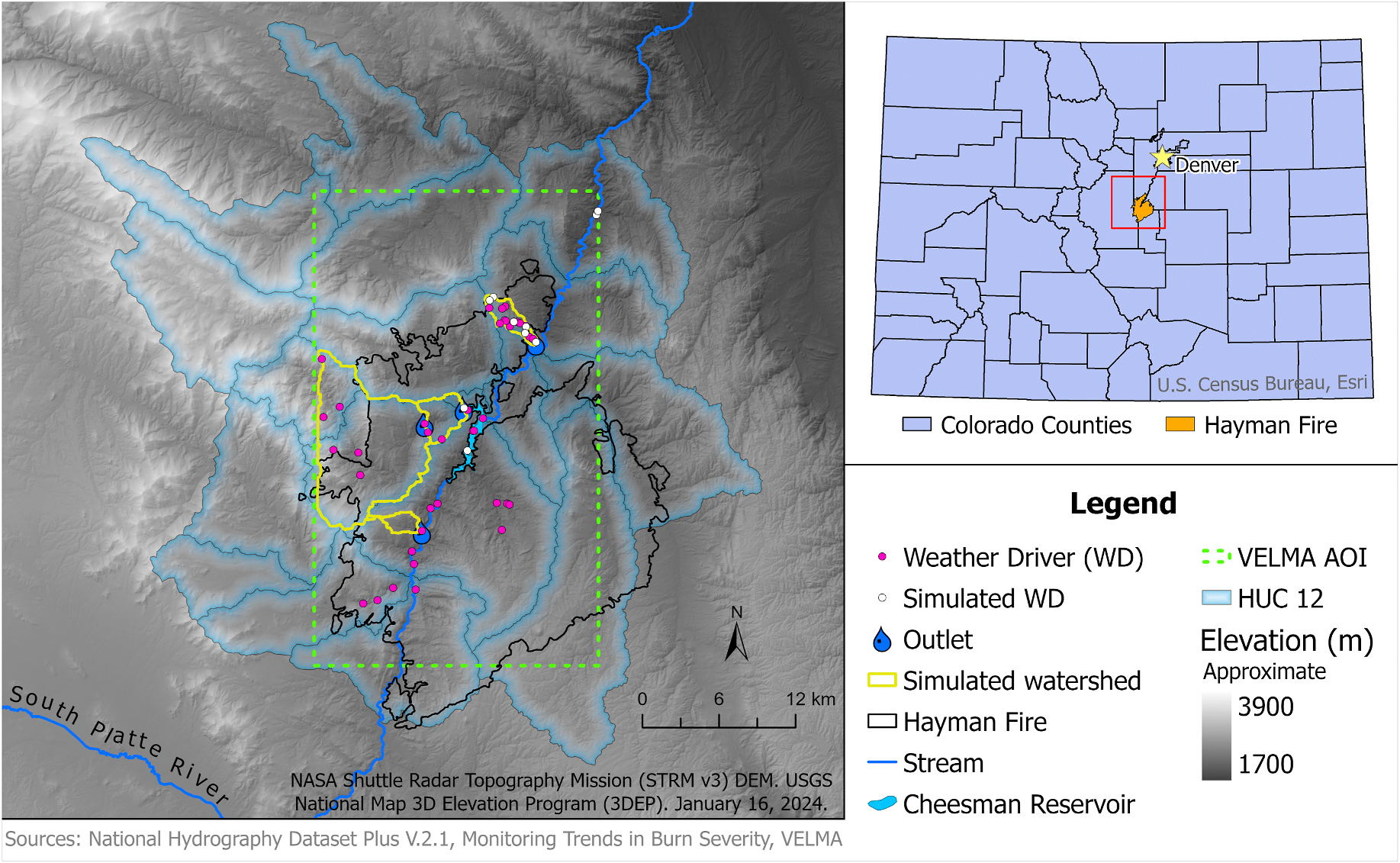
Case study area focusing on the Hayman Fire of 2002, highlighting the Brush Creek watershed (northern most one) and the other modeled watersheds using data from USEPA and USGS NHDPlus (2012).

In our case study, we focused on a rectangular AOI surrounding the Hayman Fire (Fig. 1) of 2002, southwest of Denver, which was the largest wildfire in Colorado’s state history (Wright, 2021). Centrally located in the middle of the VELMA AOI was the main channel of the South Platte River and the Cheesman Reservoir. The fire prompted the suspension of the drinking water supply associated with the Cheesman Reservoir due to water quality declines post-fire (Denver Water, 2021). This raised concerns in how to address mitigation and remediation efforts post-fire pertaining to water quality and quantity.

To assess wildfire impacts on water quality and reach dynamics, we chose the USEPA’s VELMA (McKane et al., 2014). VELMA’s ability to model daily outputs and flexibility of spatial gridded scales was optimal for investigating wildfire impacts on water quality and quantity. VELMA’s gridded eco-hydrologic pools and in-stream loss daily fluxes associated with vegetation productivity and disturbances made it an ideal model to explore biomass loss impacts within selected delineated watersheds. Since limited observed data from Brush Creek (Fig. 1) was available for comparison to modeled data, several additional acquisition points were confined within Brush Creek’s delineated catchment boundary for proof of concept. Acquisition methods were then performed across several additional locations within different HUC 12 across the VELMA AOI (Fig. 1). These data acquisition methods for VELMA, improve its performance in assessing wildfire disturbances across the area of interest (AOI). Additionally, three other watersheds, lacking validation data, were selected to represent regions impacted by the wildfire.

## 3. Data acquisition & methods

### 3.1. Data acquisition

Within our AOI, we chose to utilize the USEPA Hydrologic Micro Services (HMS) (Parmar et al., 2018) Representation State Transfer (REST) application programming interface (API) for several locations across the region. Recent scientific advancements in tandem with improvements to cyberinfrastructure, promote the ability to explore new avenues for open-source sharing of data and development within contained computational environments which incorporate FAIR (Findable, Accessible, Interoperable, Reusable) practices for data management (Choi et al., 2021). We focused our data acquisition for WDs and surface runoff from the North American Land Data Assimilation System (NLDAS) (Xia et al., 2013) reanalysis noted for its best available observations for long term climate records (Mitchell et al., 2004).

HMS (Parmar et al., 2018) provides several other climate sources inclusive of Global Land Data Assimilation System (Rodell et al., 2004), National Centers for Environmental Information (NCEI) (National Oceanic and Atmospheric Administration [NOAA], n.d.), Tropical Rainfall Measurement Mission (TRMM) (Huffman et al., 2023), and Daymet (Thornton et al., 2021), at various temporal and data formats based on user needs for a single latitude and longitude point or catchment centroid for precipitation and temperature modeled data. However, singular data collection for several locations, additional unit conversions, and formatting the raw data retrieved from the HMS data provisioning web services to initialize VELMA and other LSMs are still time-consuming and require extensive attention to detail to accurately preprocess the initialization files. Furthermore, the acquisition of collective air temperature and water budget parameters for a network of reaches or singular location is currently unavailable as an option during the export of HMS retrieved outputs.

These implications are time-sensitive when running simulations, calibration, validation, and exploratory data analysis of modeled output, especially when gridded LSMs, like VELMA, are utilized as decision support tools. For our case study AOI, NLDAS data performed the best in prior calibrations, and we will emphasize on its data retrieval methods with HMS. Additionally, for validation of surface runoff modeled in VELMA, comparisons are performed utilizing the Nash-Sutcliffe Efficiency (NSE) coefficient to determine model performance, with optimal performance occurring when the NSE is equal to 1 (Nash and Sutcliffe, 1970; Waseem et al., 2017). For performance comparison, we utilize the surface runoff data for similar acquisition periods from HMS as well as using similar automation retrieval methods performed with the WDs.

**Fig. 2.**
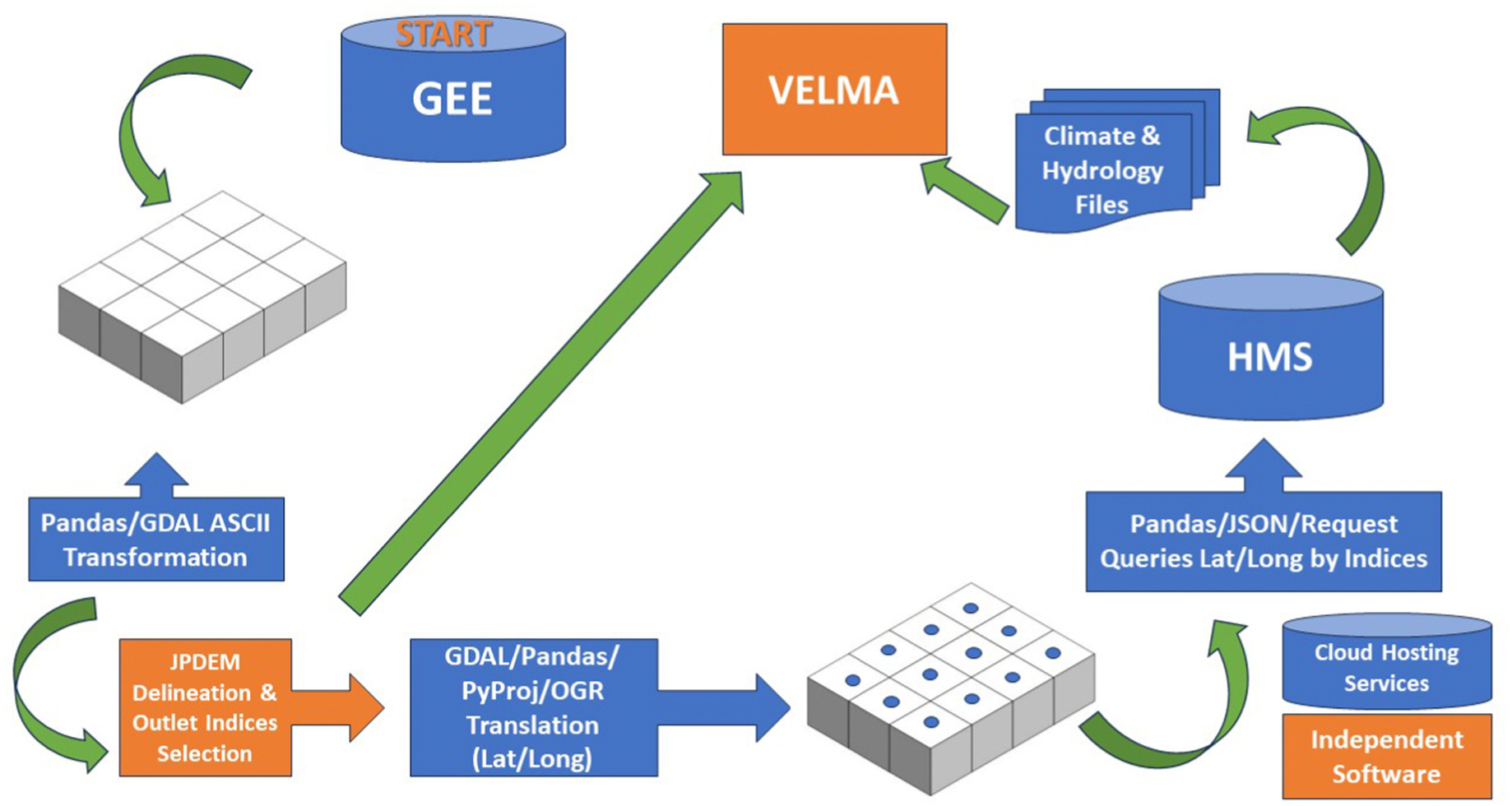
Conceptual diagram of the GEE/GDAL/HMS/VELMA workflow. Figure items in blue represent processes occurring within the Jupyter notebook kernel. Figure items in orange represent the open-sourced modeling software previously used for VELMA simulation initialization.

VELMA’s initialization requires the acquisition of a digital elevation model for a rectangular AOI (McKane et al., 2014; Pan et al., 2012). This task can be more time consuming without the knowledge and resources to execute. Using our case study AOI, focused on the Hayman Fire burn scar, we were able to create a script utilizing a rectangular polygon for the selected area of interest. Using the AOI boundary, we acquired and exported the National Aeronautics and Space Administration (NASA) Jet Propulsion Laboratory Shuttle Radar Topography Mission (SRTM) Version 3 DEM (Farr et al., 2007) through GEE. The DEM is a critical component for VELMA and any LSM as it is required for watershed delineation, water flow characterization, and assists in landscape classification of the AOI (McKane et al.; Pan et al.). Previous acquisition methods of the DEM either required stitching together tiles and additional aggregation methods tailored to a specific area of interest. This requires expertise in geospatial software which is standardly requested from the US Department of Agriculture’s (USDA) Geospatial Data Gateway (USDA, n.d.) and downloaded which consumes additional computer memory. With our methodology, the DEM acquisition is expedited and requires only the drawing of a rectangular polygon and then is reformatted from the exported GeoTiff to an ASCII (Oak Ridge National Laboratory [ORNL], n.d.) file, required for VELMA simulations. The DEM’s projected coordinates are transformed into grid cell indices utilizing spatial reference projection codes. This acquisition workflow provides greater efficiency, minimizes model initialization time, reduces manual processing errors, and facilitates reusable science for other modeling studies. The following describes the methods utilized in the automated data aggregation for 49 locations in our AOI using our automated gridded data acquisition workflow (Fig. 2).

### 3.2. Data acquisition of the DEM

**Fig. 3.**
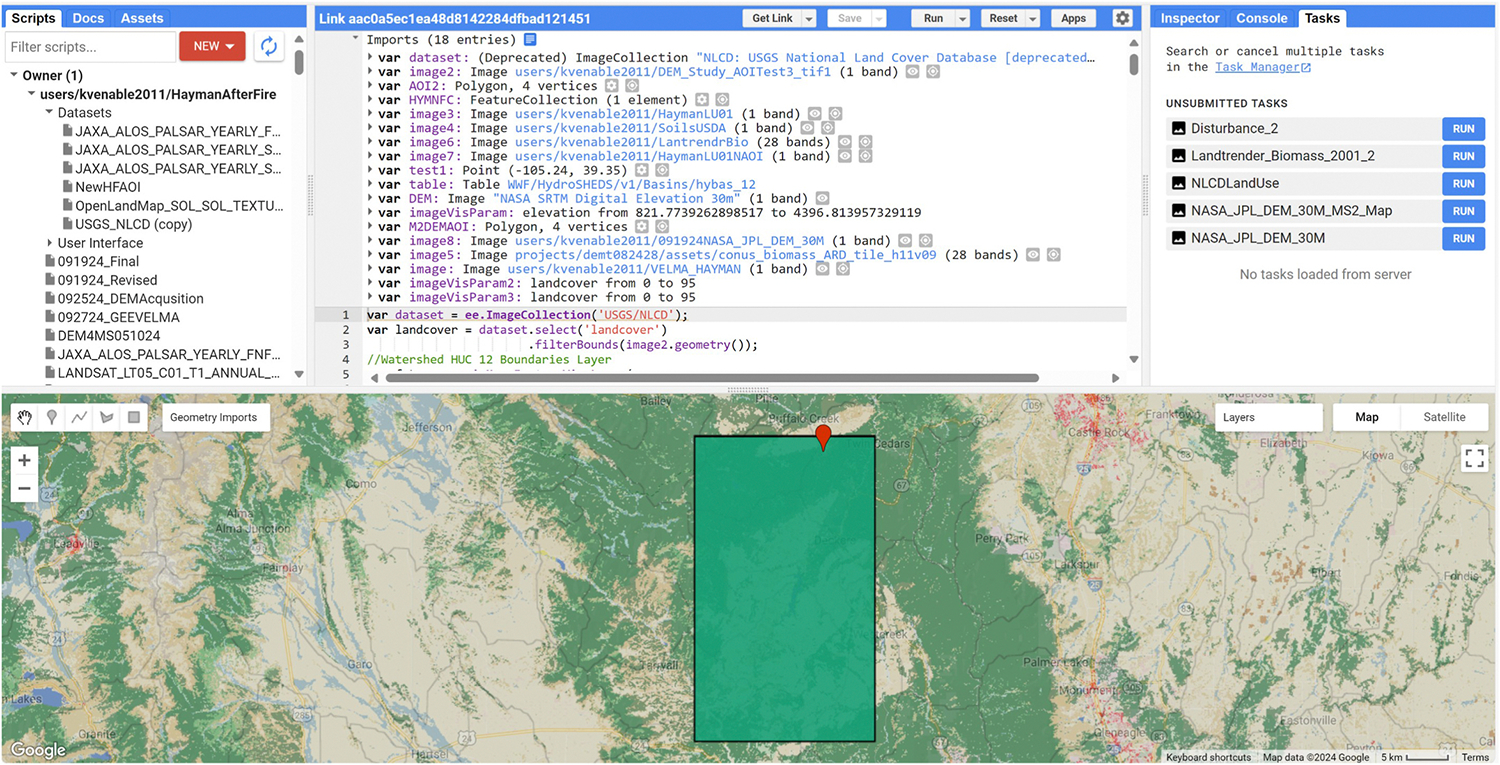
Google Earth Engine acquisition of the DEM GeoTiff.

To initialize the automation process of the WDs and surface runoff data, the acquisition of the DEM is performed. Utilizing GEE with JavaScript coding, the NASA Jet Propulsion Laboratory SRTM Version 3 DEM (Farr et al., 2007) of the entire globe is declared as a variable (Code can be found at (GEE DEM Retrieval Link). Using the rectangular drawing function in the display window, the indices of the polygon and the size of AOI are determined by the user. Using the 2001 and 2004 the United States Geological Survey (USGS) National Land Cover Database (NLCD) (Yang et al., 2018) vegetation difference shows the apparent burn scar which guided our AOI selection. The conversion of the polygon in the AOI to a feature class enables us to clip and export any dataset desired as a GeoTIFF file. While exporting the image to Google Drive, the scale is set to 30 m (best for other data collected for VELMA simulations within our AOI), and cloud optimization is utilized. Once the script is performed, output files for execution are located under the tasks tab from the right console (Fig. 3).

### 3.3. System environment dependencies

After exporting the file, we are tasked with the conversion of the GeoTiff file into an ASCII file which is required for flat processing the watershed within the Java Processing Digital Elevation Model (JPDEM) (Pan et al., 2012; McKane et al., 2014). JPDEM provides flow routing for the AOI based on an outlet or pour point. For our scenario, we choose to delineate the entire AOI’s flow routing, rather than focusing on a singular smaller catchment for analysis of wildfire impacts on water nutrient dynamics, making it easier to delineate sub-catchments from the larger AOI. We implemented a containerized approach to leverage in-line visualizations and workflow within a Jupyter notebook (Choi et al., 2021; Kluyver et al., 2016; Castronova et al., 2018). Utilizing Python, in an interactive development environment kernel, we developed an environment, named Pygdal (Erickson et al., 2013). Through conda activation of the environment and pip installation of several packages, we created an interactive notebook to preprocess and analyze the DEM and flow routing dynamics for JPDEM flat processing and VELMA simulations. This environment housed the necessary components for running GDAL (GDAL/OGR contributors, 2024), pandas, pyproj, Jupyter, matplotlib, seaborn, and the ipykernel libraries. This promoted allocating the GDAL environment through an ipykernel within a Jupyter notebook for visual display aggregation, assemblage, conversion, and exportation of all data collected and selected for the AOI analysis. (A full list of system dependencies is listed in Appendix A).

### 3.4. DEM file transformation for JPDEM initialization

**Fig. 4.**
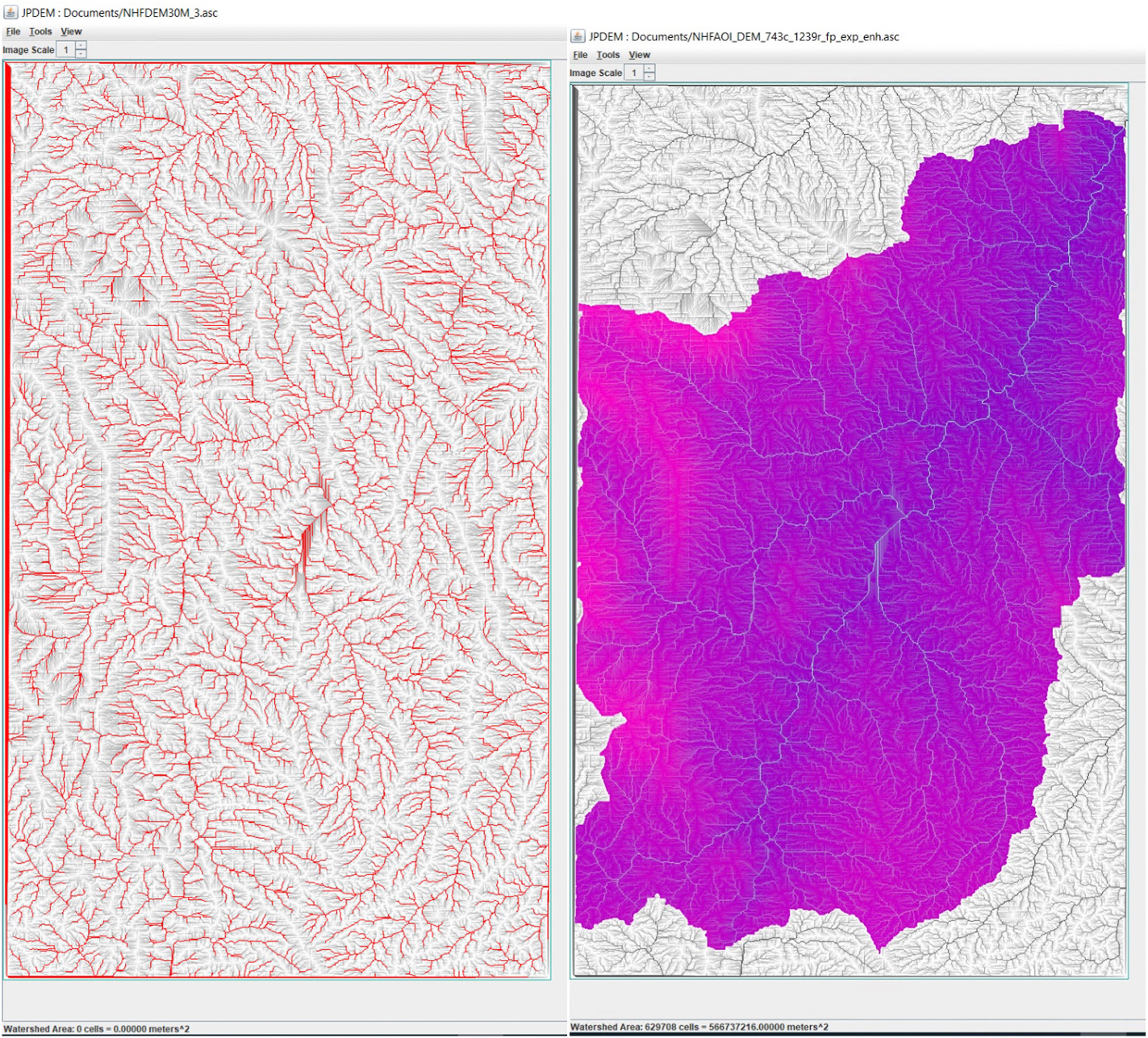
JPDEM DEM flow routing characterization (left) and watershed delineation from outlet point(right).

From the Osgeo module within Pygdal, importing GDAL has several geospatial data processing tools that are required in this workflow after exporting the DEM from GEE. There are three objectives to prepare the data for our workflow into JPDEM: 1) Convert the DEM GeoTiff file to an ASCII flat file, 2) Replacement of no data value cells (i.e., −9999), and 3) Convert the cell indices to the latitude and longitude of each cell within the AOI. To accomplish the first objective, we opened the file and utilized the GDAL warp function through its Python API. We assigned the DEM to the dataset based on user defined boundaries, well-known-text projections for the input and output files, and the geotransform of the GeoTiff. After the dataset variable declaration, we assigned the input file as the DEM GeoTiff and automated the output file naming with the ASCII file extension. Reassignment of the returned ASCII into a new dataset allows for manipulation as a DataFrame. The use of the translate function, allows us to convert the file as an XYZ file which is then read in with pandas with the comma-separated value (CSV) command. This method ensures data is not rewritten over the original source file. The new DataFrame provides the foundation to filter and replace the null DEM values with 1 and to calculate the cell indices, index numbers, and latitude and longitude coordinates (*Noted-DEM files used in JPDEM cannot have null values). After null value replacement and updating the nodata header value to −9999 through string replacement, the ASCII file is exported out and ready for use in JPDEM (Fig. 4).

### 3.5. Projected coordinate conversion

Before calculation of the converted projected coordinates to raster cell indices, we used the GetGeoTransform function to assign the header (ORNL, n.d.) information associated with the ASCII grid format. We incorporated the Transformer’s from_crs and transform functions from the pyproj (Whitaker et al., 2019) module through a variable declaration. This enables the calculation of the latitude and longitude using the x and y meter values within the projection assignment to reflect the projected coordinate systems respective to the well-known text (WKT) identifiers associated with our frame of reference (inProj = Proj (‘epsg:26913′), outProj = Proj(‘epsg:4326′)). Calculations of the row and column numbers for each cell in the AOI were updated using a script from McKane et al. (2014) which is a coordinate parser utilizing the x, y, and assigned geotransform header. The output values are appended within the existing XYZ DataFrame using pandas’ iterrows function merged through DataFrame concatenation with the series of data. The resultant DataFrame provides all the information needed for data acquisition within HMS and preprocessing for VELMA inputs.

### 3.6. JPDEM flat processing DEM for AOI

Following the VELMA user guide (McKane et al., 2014), we used the DEM ASCII file and chose the flat processing mode and delineated the watershed based on the pour point. Within JPDEM, outlet delineation points and indices of interest must correlate with the row, column, and indices of the developed DataFrame. Suppose the user chooses to delineate the AOI or its sub-catchments. In those cases, JPDEM outputs additional files inclusive of the smaller catchments which distinguish values of 0 for locations found in the watershed, 1 for the delineation or outlet point, and −1 value for areas outside of the delineation. These identifiers within the sub-catchments easily enables masking the DataFrame to select WDs and surface runoff values found within the AOI for a particular smaller catchment area. Usage of the iloc pandas function allows parsing indices within the larger AOI context. Sampling and querying locations are now present within the DataFrame.

In our case study, we chose to identify locations that were randomly selected based on the elevation gradient to diversify WDs of the front range mountains. We chose to focus on a small creek named Brush Creek, due to the availability of pre- and post-fire observations for our delineated sub-catchment within the larger AOI and other locations of interest across the AOI. The exportation of the indices of the selected cells is performed by cross-referencing the query of these chosen cell indices into the DataFrame allowing ease in spatial identification of the location within the map. A feasible option to view indices, may be to create a point variable (test1, Fig. 3) within the DEM GEE acquisition window and place the latitude/longitude coordinates within the variable declaration to view the position of the indices you have selected. Several flat processing types are available within JPDEM and may produce modeled flow dynamics differently and vary in execution time based on your AOI’s size, spatial dynamics, and cell size declaration (Fig. 4).

### 3.7. Initialization results with HMS REST API

#### 3.7.1. Flexible acquisition locations with key-value pairs

After acquiring the cells indices, latitude, and longitude within the DataFrame, we can use the HMS REST API to retrieve singular WDs or a batch of locations. The locations within a loop, are iterated and queried across a list of outlet points, to acquire the data necessary to initialize the VELMA simulations. Acquisition and preprocessing methods for climate records within VELMA can be performed with a single WD (default model) or a network of WDs (spatial weather model) across the delineated AOI or sub-catchment. We performed this process using the datetime, numpy, pandas, requests, and JavaScript Object Notation (JSON) Python libraries within the environment.

Use of JPDEM’s flat processed DEM applying the DEM flow test and delineation tool allows exportation of the selected list of outlets or cell indices providing a working file that can be read into the notebook to parse and collect the data. The delineated outlet point(s) indices gridded values are exported inclusive of watershed boundary. Variable declaration of the start year for the data available and creation of the ending date year are developed from the usage of the date range function setting the periods equivalent to the number of years. For our case study, NLDAS availability was present for 28 years. To chunk the data into smaller portions for acquisition through the API and prevent bottlenecking, annual data was collected for each cell before reaggregation which is nested in the outer loop for the data collection.

**Fig. 5.**
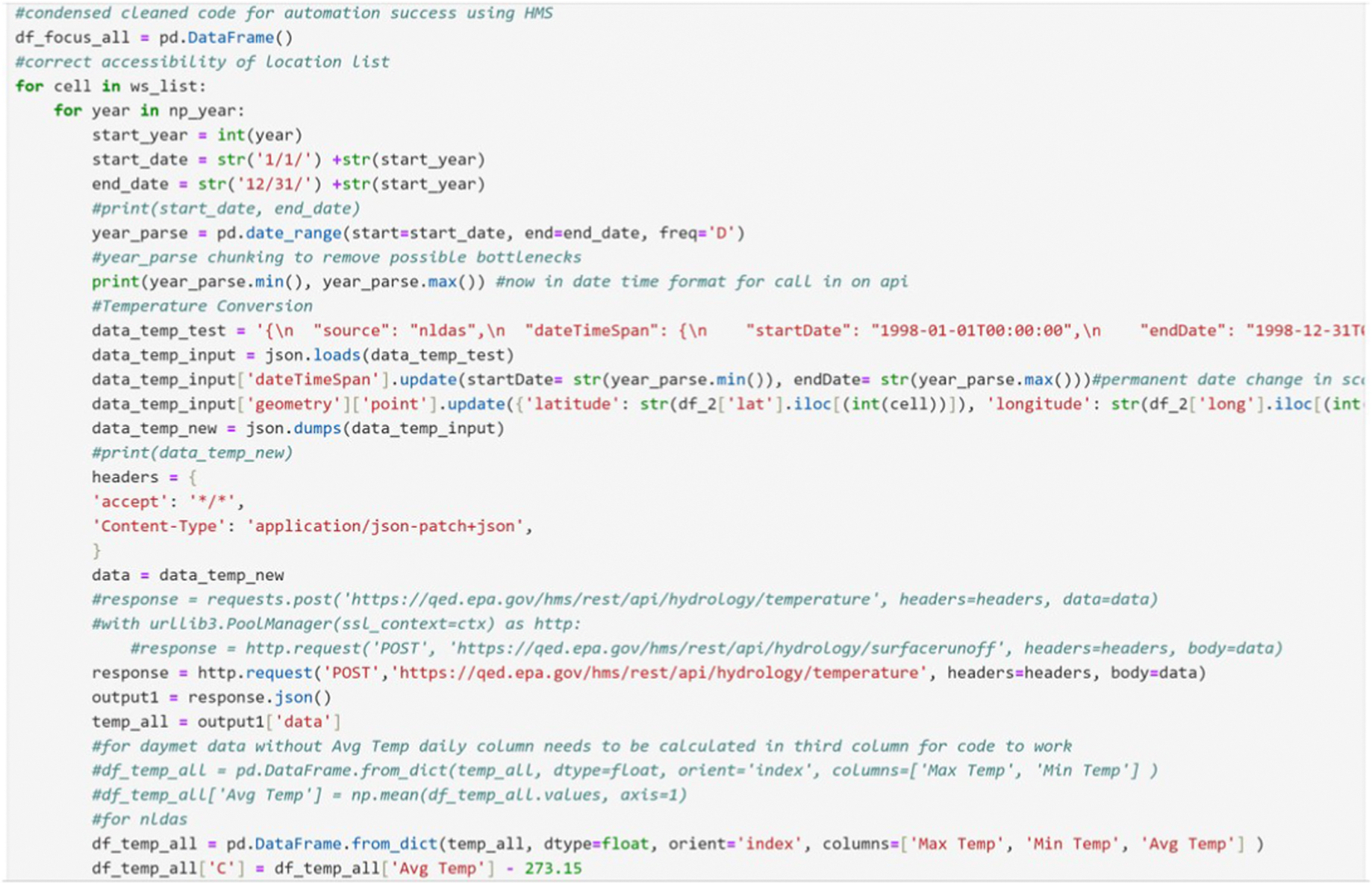
Code snippet for nested loop iteration for selected outlet points for temperature using the HMS REST API.

The declaration of the start and end date as a string allows updates to the acquisition period from the values in the outer loop. First, we develop an empty DataFrame and then select the complete year of dates for which data are acquired from HMS. The use of the JSON loads and update functions allows for updating the API key-value pairs to change the date, geometry point’s latitude, and longitude values based on the cell indices. This updated the JSON text for the API data and secondly acquired the temperature data for the period in question. This data string of key-value pairs was passed into the response request. The output was exported as a JSON response and queried from the data output as a dictionary (Fig. 5).

#### 3.7.2. DataFrame iteration development for VELMA initialization

**Fig. 6.**
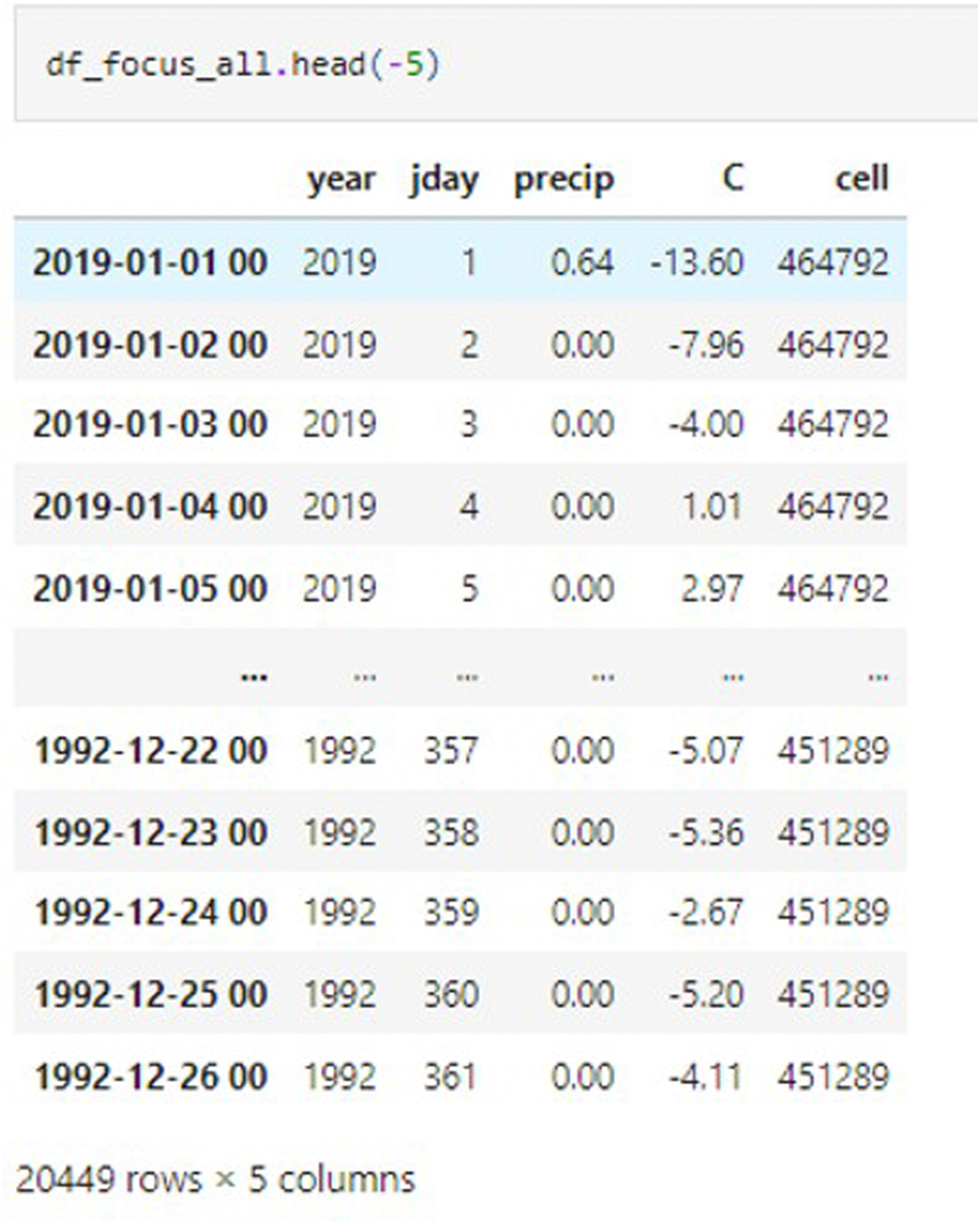
Concatenated from HMS REST API data acquisition required for VELMA initialization for spatial weather model.

The resulting data dictionary was converted into a DataFrame, in addition to the conversion of the temperature from Kelvin to Celsius. The focused annual DataFrame provided a parsable date indexing, year, Julian day, and cell indicies enhancing querying capabilities (Fig. 6).

Similar data retrieval processes were performed for the precipitation data using the HMS REST API request. With the data obtained, the precipitation data were copied into the annually focused DataFrame. After closure of the annual loop’s acquisition, the data were concatenated with the prior existing empty DataFrame adding the new data information for each year of acquisition after each iteration. VELMA’s two weather model choices require different formatting for use within the model. Before exporting the DataFrame for the collected observations, it is sorted by date. To export the data, we developed an autonomous naming convention that accounts for the current date, data acquisition source, acquisition period, and cell index for the unique filename. Using the cell list of indices, we perform loop iterations to create individual files queried by cell indices within the DataFrame. These cells are written to the computer system by importing the os package.

To initialize VELMA’s single station (default) and spatial weather models, we developed two aggregation methods for data preprocessing to meet the file input requirements. The single station weather model needs both individual daily precipitation and temperature files with one value for each day during the period of the model simulations. The spatial weather model has additional requirements including the year, Julian day, precipitation (mm), and average air temperature (degrees C). Furthermore, we developed another file which contains the WD’s information and automated filename for each index required including the row, column, and cell index number. During the DataFrame export as a CSV, we simply remove the headers from the files. Our code allows for the ease of choosing your indices and prepopulating these files necessary for the desired simulation method. Additional advancements and similar methods were utilized to retrieve daily surface runoff rates as well with the HMS REST API.

## 4. Results

We have created a repository including a Jupyter Notebook and data outputs surrounding the GEE/GDAL/HMS/VELMA acquisition workflow integration. This code assists with the formatting and acquisition of gridded data products including the DEM, climate, and hydrologic data necessary for initialization in VELMA or other LSMs. Utilizing the DEM’s grid cell indices and translated latitude and longitude coordinates, we can easily query the outlets or points of interest within a watershed from JPDEM within a rectangular AOI. With this gridded network translation, we can quickly acquire climate, hydrologic, and terrain data from a variety of sources for anywhere in the US. Users are allowed to obtain random samples of indices or create curated lists of locations without extreme computing power and rely solely on open-source software. The addition of graphical libraries within the development environment, provides a place to conduct exploratory data analysis through matplotlib and seaborn. Our efforts assist in acquisition of large collections of data for multiple locations within gridded data products to initialize LSMs without the knowledge of understanding a coding language encompassing a “low code/no code” approach (Di Ruscio et al., 2022).

Because of the REST API accessibility, workflow users can obtain large amounts of data for multiple locations and large time spans (>20 years) from several modeled datasets. This enables users to perform these tasks without having the computing power, tools, and knowledge to disseminate and use within VELMA and other LSMs. This rapid increase in data acquisition and preprocessing efficiency reduces initializing time with LSMs but is specifically tailored for VELMA initialization inputs. Use of the timeit magic functions across only the surface runoff data indicate an annual loop for two indices had an acquisition rate of 543 ns ± 420 ns per loop (mean ± std. dev. of 7 runs, 1 loop each) with a 32-year acquisition test.

Due to VELMA’s flat file input requirements and versatility of the code within the DataFrame, it allows for ease of incorporation of other adaptations for other models requiring similar metrics. The developed repository of data allows accessibility for others performing socio-environmental impact studies within the Front Range. The Jupyter Notebook walks through the DEM transformation and data acquisition for each parameter prior to exporting using a unique recursive file naming system. The repository and acquisition notebook can be found at (https://github.com/USEPA/HMS-Gridded-Automation).

**Fig. 7.**
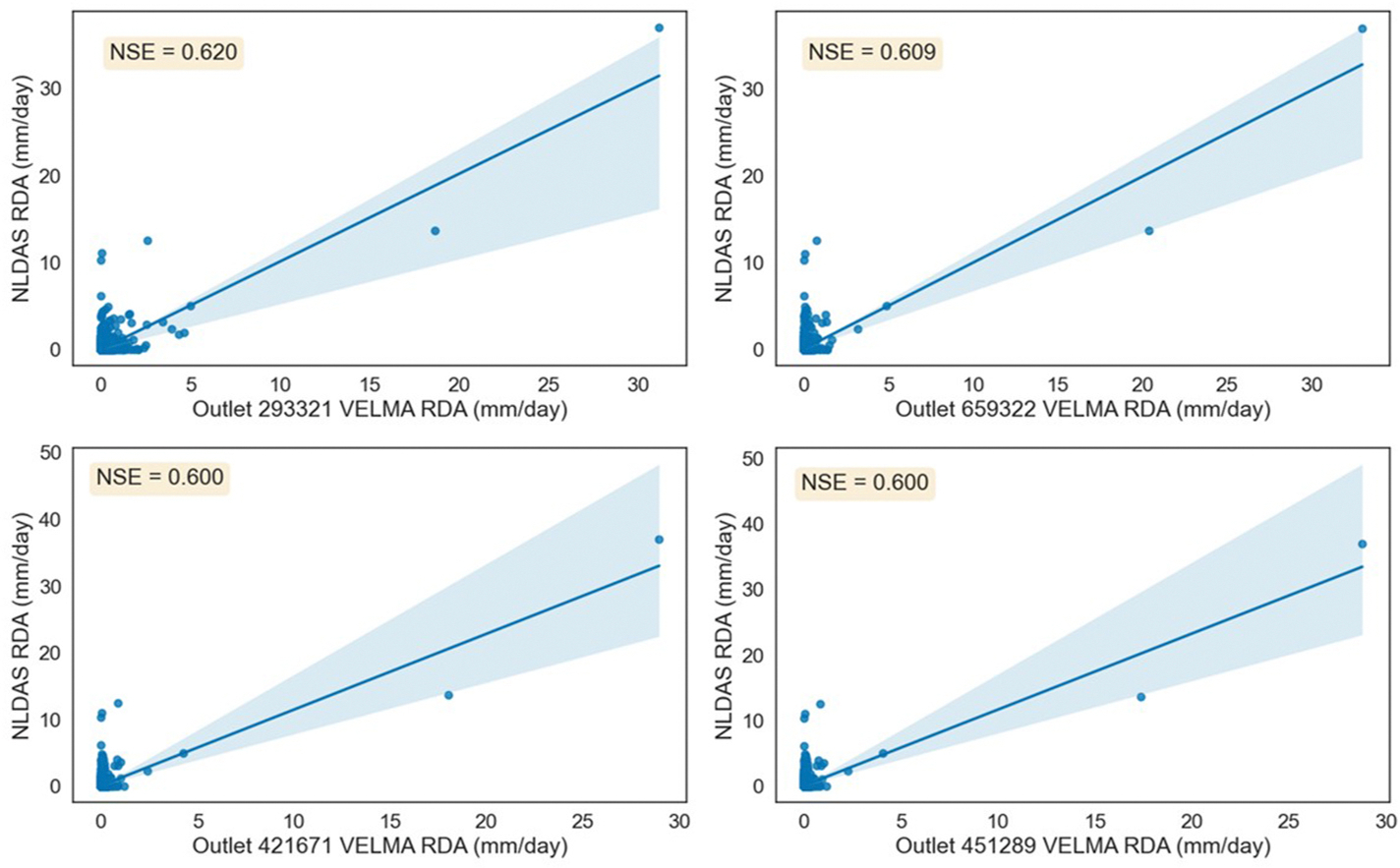
Scatterplot and trendline comparison of four modeled runoff delineated averages (RDA) of watershed outlets within the AOI during 2000–2006 against NLDAS Brush Creek surface runoff (Outlet 293321) or Outlet 39377 with Nash-Sutcliffe Efficiency (NSE) coefficents. Shaded areas depict the 95 percentile confidence interval.

Using this workflow developed for VELMA, there were several advancements performed. First, we were able to accelerate the number of simulations conducted. Secondly, we easily expanded our spatial WD network for more coverage across the AOI. Additional calibration metrics and data sources used to initialize this model found are discussed in Venable et al. (2024). This assimilation of new data enabled better calibration performance across several outlets of delineated watersheds using VELMA’s multimode function. In comparison to NLDAS’s Brush Creek modeled daily runoff values, we’ve highlighted four watersheds (Fig. 1). Simulated results yielding good NSE calibrations according to Nash and Sutcliffe (1970), Waseem et al. (2017), and Venable et al. (Fig. 7). These VELMA simulations were performed utilizing VELMA’s multimode with two scenarios depicting 11 spatial WDs used across the AOI (Fig. 1) and 3 spatial WDs within Brush Creek. VELMA modeled results correlated best during low flows in comparison to NLDAS. The best calibration occurred along Brush Creek (Outlet 293321), which had extensive testing and recalibration as discussed in Venable et al. The performance of other modeled watersheds originated from Brush Creek’s initialization calibration metrics.

## 5. Discussion

The novelty of this workflow is that it curates copious amounts of data relevant to a variety of research efforts without the hassles of having a working knowledge of geospatial software and code development. This is necessary for the advancement of analyses, decision support efforts, and reduced time for VELMA and additional LSMs initialization. Acquisition and curation of data requires extensive time, knowledge, and research. Moreover, cultivating stakeholder engagement through access and interaction with easily understandable models can yield better policy design and interventions (Asif et al., 2023). Recent advancements in earth observations requires improvements in methodologies for Earth science and diversifies opportunities in environmental research (Guo et al., 2016). Our GEE/GDAL/HMS/VELMA workflow provides an open access solution to obtain, aggregate, examine, and export environmental products for researchers who deploy and evaluate gridded LSMs.

Additional adaptions of this code include batch collection of data with COMIDs supporting NHDPlus (USEPA & USGS, 2012) networks and examination of hydrologic unit codes (HUCs) based on users’ needs. We also acquired National Water Model streamflow retrospective data (NOAA, 2023) from the HMS REST API through similar batch acquisition methods listed previously. This workflow can connect with graphical user interfaces (GUI) and dashboards with multiple locations as data availability of gridded products increases over time with the advancement of unmanned aerial vehicles and satellite data products.

This platform employing gridded networks of data provide quantitative connections between a variety of other environmental and socio-economic data at similar scales. HMS has other key values that can be updated within the API request as well, including evapotranspiration and soil moisture, dependent on the model of choice. It is noted that additional data aggregation methods must be customized due to differences in modeled representation of traditional Julian calendars as with Daymet (Thornton et al., 2021) data. We had the opportunity to populate plots for comparisons through this automation as well with adaptations to the output file naming conventions for the image exports. Due to recent transitions within the rest API server, we needed to provide an additional client handler to access the data. We chose the urllib3 pool manager with the http response request post to resolve data handling issues, which may not be required after the server transition has been completed. When resolved, we can deploy a traditional JSON request through the HMS REST API.

There are endless opportunities for this workflow through the development of dashboards and GUIs for socio-economic research with minimal cost, labor, or time. Interactive notebooks like Jupyter notebook enable easily sharing with others in a readable format outside of the integrated development environment platform. If continuous data retrieval efforts are made, development of a working database for searchable data without using cloud computing resources is possible if queries are continually logged into one working repository, like HydroShare (Choi et al., 2021). This, however, can require extensive storage resources. It is also important to maintain the virtual environment’s version control for GDAL (3.6.3), pyproj (3.4.1) and Python (3.11.10) for this workflow as reference projection transformers must be obtained in later versions. The research presented provides two shareable interactive notebooks which guide the pre- and post-processing data acquisition from GEE and HMS, including a unique file naming system for supporting initialization files supported by VELMA and other LSMs.

## 6. Conclusion

In conclusion, this GEE/GDAL/HMS/VELMA acquisition workflow within a Jupyter Notebook provides greater efficiency, minimizes model initialization time, reduces manual processing errors, and facilitates reusable methodology for other modeling studies. With our data collection efforts, we developed a database of usable modeled NLDAS climate records for almost 50 locations across our AOI located in the mountainous terrain of the Front Range in Colorado. Furthermore, with the use of GDAL transformation function, projected coordinates of grid cell indices, latitude, and longitude are performed through a recursive update to the JSON response requests using the HMS REST API.

Prior training and simulation guidance suggested using GIS and Parameter-elevation Regressions on Independent Slopes Model PRISM (PRISM Group, 2025) or Daymet (Thornton et al., 2021) data for VELMA initialization and took technical skill to determine cell indices with a specific latitude and longitude, thus making it more tedious to determine the physical representation of the gridded indices. Now, it is easy to obtain a natural mapping schema with the degree coordinates of the locations. With the use of the workflow developed, we accelerated the number of simulations conducted across the AOI, and increased calibration and validation of simulations which will be detailed in future work. Our workflow allows novice users to explore, customize to their area of interest, and automate data acquisition, preprocessing, and aggregations, with automatic naming conventions for unique file names used for initialization within VELMA. This advancement will encourage more users to easily deploy VELMA and other LSMs, thus accelerating implementation of aggregated gridded data products as decision support tools.

## Data Availability

Software and data repository can be found at https://github.com/USEPA/HMS-Gridded-Automation.

## Appendix A: Software List

**Table:**

Name	Version	Build	Channel
asttokens	2.4.1	pyhd8ed1ab_0	conda-forge
blosc	1.21.6	h85f69ea_0	conda-forge
boost-cpp	1.78.0	h9f4b32c_4	conda-forge
bzip2	1.0.8	h2466b09_7	conda-forge
ca-certificates	2024.8.30	h56e8100_0	conda-forge
cairo	1.16.0	h0ac17fb_1012	conda-forge
certifi	2024.8.30	pyhd8ed1ab_0	conda-forge
cfitsio	4.2.0	h9ebe7e4_0	conda-forge
colorama	0.4.6	pyhd8ed1ab_0	conda-forge
comm	0.2.2	pyhd8ed1ab_0	conda-forge
curl	8.10.1	h1ee3ff0_0	conda-forge
debugpy	1.8.5	py311hda3d55a_1	conda-forge
decorator	5.1.1	pyhd8ed1ab_0	conda-forge
exceptiongroup	1.2.2	pyhd8ed1ab_0	conda-forge
executing	2.1.0	pyhd8ed1ab_0	conda-forge
expat	2.6.3	he0c23c2_0	conda-forge
font-ttf-dejavu-sans-mono	2.37	hab24e00_0	conda-forge
font-ttf-inconsolata	3	h77eed37_0	conda-forge
font-ttf-source-code-pro	2.038	h77eed37_0	conda-forge
font-ttf-ubuntu	0.83	h77eed37_2	conda-forge
fontconfig	2.14.2	hbde0cde_0	conda-forge
fonts-conda-ecosystem	1	0	conda-forge
fonts-conda-forge	1	0	conda-forge
freetype	2.12.1	hdaf720e_2	conda-forge
freexl	1.0.6	h67ca5e6_1	conda-forge
gdal	3.6.3	py311h4bd9738_1	conda-forge
geos	3.11.2	h1537add_0	conda-forge
geotiff	1.7.1	hb4c6682_7	conda-forge
gettext	0.22.5	h5728263_3	conda-forge
gettext-tools	0.22.5	h5a7288d_3	conda-forge
hdf4	4.2.15	h1334946_6	conda-forge
hdf5	1.12.2	nompi_h57737ce_101	conda-forge
icu	70.1	h0e60522_0	conda-forge
importlib-metadata	8.5.0	pyha770c72_0	conda-forge
intel-openmp	2024.2.1	h57928b3_1083	conda-forge
ipykernel	6.29.5	pyh4bbf305_0	conda-forge
ipython	8.27.0	pyh7428d3b_0	conda-forge
jedi	0.19.1	pyhd8ed1ab_0	conda-forge
jupyter_client	8.6.3	pyhd8ed1ab_0	conda-forge
jupyter_core	5.7.2	py311h1ea47a8_0	conda-forge
kealib	1.5.0	h61be68b_0	conda-forge
krb5	1.21.3	hdf4eb48_0	conda-forge
lcms2	2.15	h3e3b177_1	conda-forge
lerc	4.0.0	h63175ca_0	conda-forge
libaec	1.1.3	h63175ca_0	conda-forge
libasprintf	0.22.5	h5728263_3	conda-forge
libasprintf-devel	0.22.5	h5728263_3	conda-forge
libblas	3.9.0	24_win64_mkl	conda-forge
libcblas	3.9.0	24_win64_mkl	conda-forge
libcurl	8.10.1	h1ee3ff0_0	conda-forge
libdeflate	1.17	hcfcfb64_0	conda-forge
libexpat	2.6.3	he0c23c2_0	conda-forge
libffi	3.4.2	h8ffe710_5	conda-forge
libgdal	3.6.3	hd256549_1	conda-forge
libgettextpo	0.22.5	h5728263_3	conda-forge
libgettextpo-devel	0.22.5	h5728263_3	conda-forge
libglib	2.78.1	he8f3873_0	conda-forge
libhwloc	2.11.1	default_h8125262_1000	conda-forge
libiconv	1.17	hcfcfb64_2	conda-forge
libintl	0.22.5	h5728263_3	conda-forge
libintl-devel	0.22.5	h5728263_3	conda-forge
libjpeg-turbo	2.1.5.1	hcfcfb64_1	conda-forge
libkml	1.3.0	h538826c_1021	conda-forge
liblapack	3.9.0	24_win64_mkl	conda-forge
libnetcdf	4.9.1	nompi_h83fa41b_102	conda-forge
libpng	1.6.44	h3ca93ac_0	conda-forge
libpq	15.8	hf13906b_0	conda-forge
librttopo	1.1.0	he1da8c1_13	conda-forge
libsodium	1.0.20	hc70643c_0	conda-forge
libspatialite	5.0.1	h2bb424f_24	conda-forge
libsqlite	3.46.1	h2466b09_0	conda-forge
libssh2	1.11.0	h7dfc565_0	conda-forge
libtiff	4.5.0	hc3b8658_5	conda-forge
libwebp-base	1.4.0	hcfcfb64_0	conda-forge
libxml2	2.12.7	h0f24e4e_4	conda-forge
libzip	1.10.1	h1d365fa_3	conda-forge
libzlib	1.3.1	h2466b09_1	conda-forge
lz4-c	1.9.4	hcfcfb64_0	conda-forge
matplotlib-inline	0.1.7	pyhd8ed1ab_0	conda-forge
mkl	2024.1.0	h66d3029_694	conda-forge
nest-asyncio	1.6.0	pyhd8ed1ab_0	conda-forge
numpy	1.26.4	py311h0b4df5a_0	conda-forge
openjpeg	2.5.0	ha2aaf27_2	conda-forge
openssl	3.3.2	h2466b09_0	conda-forge
packaging	24.1	pyhd8ed1ab_0	conda-forge
parso	0.8.4	pyhd8ed1ab_0	conda-forge
pcre2	10.40	h17e33f8_0	conda-forge
pickleshare	0.7.5	py_1003	conda-forge
pip	24.2	pyh8b19718_1	conda-forge
pixman	0.43.4	h63175ca_0	conda-forge
platformdirs	4.3.6	pyhd8ed1ab_0	conda-forge
poppler	23.03.0	h934c637_1	conda-forge
poppler-data	0.4.12	hd8ed1ab_0	conda-forge
postgresql	15.8	hf13906b_0	conda-forge
proj	9.1.1	heca977f_2	conda-forge
prompt-toolkit	3.0.47	pyha770c72_0	conda-forge
psutil	6.0.0	py311he736701_1	conda-forge
pthreads-win32	2.9.1	hfa6e2cd_3	conda-forge
pure_eval	0.2.3	pyhd8ed1ab_0	conda-forge
pygments	2.18.0	pyhd8ed1ab_0	conda-forge
pyproj	3.4.1	py311h36482e4_1	conda-forge
python	3.11.10	hce54a09_0_cpython	conda-forge
python-dateutil	2.9.0	pyhd8ed1ab_0	conda-forge
python_abi	3.11	5_cp311	conda-forge
pywin32	306	py311h12c1d0e_2	conda-forge
pyzmq	26.2.0	py311h484c95c_2	conda-forge
setuptools	73.0.1	pyhd8ed1ab_0	conda-forge
six	1.16.0	pyh6c4a22f_0	conda-forge
snappy	1.2.1	h23299a8_0	conda-forge
sqlite	3.46.1	h2466b09_0	conda-forge
stack_data	0.6.2	pyhd8ed1ab_0	conda-forge
tbb	2021.13.0	hc790b64_0	conda-forge
tiledb	2.13.2	h3132609_0	conda-forge
tk	8.6.13	h5226925_1	conda-forge
tornado	6.4.1	py311he736701_1	conda-forge
traitlets	5.14.3	pyhd8ed1ab_0	conda-forge
typing_extensions	4.12.2	pyha770c72_0	conda-forge
tzdata	2024a	h8827d51_1	conda-forge
ucrt	10.0.22621.0	h57928b3_0	conda-forge
uriparser	0.9.8	h5a68840_0	conda-forge
vc	14.3	h8a93ad2_21	conda-forge
vc14 runtime	14.40.33810	ha82c5b3_21	conda-forge
vs2015 runtime	14.40.33810	h3bf8584_21	conda-forge
wcwidth	0.2.13	pyhd8ed1ab_0	conda-forge
wheel	0.44.0	pyhd8ed1ab_0	conda-forge
xerces-c	3.2.5	he0c23c2_1	conda-forge
xz	5.2.6	h8d14728_0	conda-forge
zeromq	4.3.5	he1f189c_5	conda-forge
zipp	3.20.2	pyhd8ed1ab_0	conda-forge
zlib	1.3.1	h2466b09_1	conda-forge
zstd	1.5.6	h0ea2cb4_0	conda-forge

## Abbreviations:

AOI: Area of Interest
ASCII: American Standard Code for Information Interchange
API: Application Programming Interface
CSV: comma-separated value
DDDAS: dynamic data driven application system
DEM: Digital Elevation Model
USEPA: United States Environmental Protection Agency
FAIR: Findability, Accessibility, Interoperability, and Reusability
GDAL: Geospatial Data Abstraction Library
GEE: Google Earth Engine
GeoTIFF: Geographic Tagged Image File Format
GLDAS: Global Land Data Assimilation System
GUI: Graphical User Interface
HMS: Hydrologic Micro Services
HUC: hydrologic unit code
JPDEM: Java Processing Digital Elevation Model
JSON: Java Script Object Notation
LANDIS: LANDscape DIsturbance and Succession
LSMs: Land Surface Models
NASA: National Aeronautical and Space Administration
NCEI: National Centers for Environmental Information
NLCD: National Land Cover Database
NLDAS: North American Land Data Assimilation System
NOAA: National Oceanic and Atmospheric Administration
NSE: Nash-Sutcliffe Efficiency
ORNL: Oak Ridge National Laboratory
PRISM: Parameter-elevation Regressions on Independent Slopes Model
REST: Representation State Transfer
SRTM: Shuttle Radar Topography Mission
TRMM: Tropical Rainfall Measuring Mission
US: United States
VELMA: Visualizing Ecosystem and Land Management Assessment
WD: Weather Drivers
WKT: well-known text
